# Myxobacterial Genomics and Post-Genomics: A Review of Genome Biology, Genome Sequences and Related ‘Omics Studies

**DOI:** 10.3390/microorganisms9102143

**Published:** 2021-10-13

**Authors:** David E. Whitworth, Natashia Sydney, Emily J. Radford

**Affiliations:** Institute of Biological, Environmental and Rural Sciences, Aberystwyth University, Aberystwyth SY23 3DD, UK; nas39@aber.ac.uk (N.S.); emr33@aber.ac.uk (E.J.R.)

**Keywords:** comparative genomics, functional genomics, genome evolution, genome organisation, pan-genome, proteomics, taxonomy, transcriptomics

## Abstract

Myxobacteria are fascinating and complex microbes. They prey upon other members of the soil microbiome by secreting antimicrobial proteins and metabolites, and will undergo multicellular development if starved. The genome sequence of the model myxobacterium *Myxococcus xanthus* DK1622 was published in 2006 and 15 years later, 163 myxobacterial genome sequences have now been made public. This explosion in genomic data has enabled comparative genomics analyses to be performed across the taxon, providing important insights into myxobacterial gene conservation and evolution. The availability of myxobacterial genome sequences has allowed system-wide functional genomic investigations into entire classes of genes. It has also enabled post-genomic technologies to be applied to myxobacteria, including transcriptome analyses (microarrays and RNA-seq), proteome studies (gel-based and gel-free), investigations into protein–DNA interactions (ChIP-seq) and metabolism. Here, we review myxobacterial genome sequencing, and summarise the insights into myxobacterial biology that have emerged as a result. We also outline the application of functional genomics and post-genomic approaches in myxobacterial research, highlighting important findings to emerge from seminal studies. The review also provides a comprehensive guide to the genomic datasets available in mid-2021 for myxobacteria (including 24 genomes that we have sequenced and which are described here for the first time).

## 1. Myxobacterial Genomics

Myxobacteria are ubiquitous soil-dwelling bacteria which have attracted considerable research interest due to their complex behaviours, ecological importance and production of potentially useful bio-active compounds [1,2]. They have rod-shaped cells, typically 0.5 microns in width by 3–8 microns in length, which can move backwards and forwards over a surface, in the direction of the long axis of the cell. Myxobacteria are found abundantly and ubiquitously in soils, but have also been found in virtually all other environments where they have been looked for. As apex microbial predators, they are able to kill and feed on the other microbes they encounter. Prey killing is achieved by the secretion of antimicrobial metabolites and digestive enzymes into the public commons, which cause prey cell lysis [3,4,5]. When prey and nutrients are scarce, myxobacteria instead undergo cooperative multicellular development. A population of 100,000 cells orchestrate their movements to aggregate together into fruiting bodies. Some species make fruiting bodies which are simple raised mounds, while other myxobacteria make complex tree-shaped structures. Within fruiting bodies, a subset of cells differentiate into metabolically dormant and stress-resistant myxospores [6], which germinate when nutrients/prey become available again.

The most thoroughly studied myxobacterium is *Myxococcus xanthus* DK1622, which has been investigated since the 1970s [7]. Over the following decades, a genetic toolbox was developed for DK1622 and other *M. xanthus* strains (including transposons, reporter genes, targeted mutagenesis systems, transducing phage, and gene insertions), which enabled elucidation of the genetic basis of fruiting body development and other myxobacterial behaviours [8,9]. 

Prior to genome sequencing, it was already known that myxobacteria typically had large genomes [10,11], and that their genomes had a high (~70%) %GC content [12,13] (the percentage of G–C base pairs in the genome). Later, cloning and sequencing of individual genes confirmed the GC-rich nature of myxobacterial genomes, and also observed reading frame bias in protein-coding genes [14,15]. To maintain an overall %GC in protein-coding sequences (CDSs), the three bases of the triplet codon have distinctive %GC biases, and the pattern of those biases can be helpful when identifying protein-coding features in genome sequences [16].

### 1.1. The Genome Sequence of M. xanthus DK1622

The first myxobacterial genome sequence to be made publicly available was that of *Anaeromyxobacter dehalogenans* 2CP-C [17]. However, the model myxobacterium DK1622 was the first myxobacterium to have its genome sequence described in the literature [18]. Sequencing of *M. xanthus* DK1622 was initially performed by Monsanto, producing a draft genome known as the M1 genome, which remains unpublished. Using the M1 genome, complete sequencing was then undertaken by The Institute of Genome Research (TIGR), and annotation of the resulting genome sequence was undertaken as a project involving the entire myxobacteria research community.

When sequenced, *M. xanthus* DK1622′s exact genome size was revealed to be 9.14 Mbp, comprised of a single circular chromosome with a %GC content of 68.9. The large size of the genome compared with other bacteria was proposed to have been due in part to lineage-specific duplications generating paralagous genes [18]. Later, Huntley et al. [19] investigated the possibility that the large size of fruiting myxobacterial genomes was due to whole genome duplication, but found no evidence for that scenario, instead reinforcing the importance of gene duplications. Such duplications seemed to have disproportionately affected genes encoding signalling proteins (Ser/Thr protein kinases, two-component signal transduction systems), transcriptional regulators, chemosensory and motility proteins. It was suggested that the increase in regulatory potential from duplications of genes encoding signalling proteins and regulators, allowed the evolution of the complex regulatory network necessary for multicellular fruiting body formation [18]. 

Using BLAST, Goldman et al. [18] also found that one-third of the CDSs of *M. xanthus* DK1622 were most similar to genes from distantly related organisms (outside class Deltaproteobacteria, which myxobacteria were assigned within at the time). In a similar study into *Bdellovibrio bacteriovorus* (a non-myxobacterial predatory bacterium classified within Deltaproteobacteria at the time), such a pattern of relatedness was taken as evidence a CDS had been acquired by horizontal gene transfer (HGT) from a phylogenetically distant prey organism [20].

The genome sequence of *M. xanthus* DK1622 also revealed the presence of large gene clusters for the synthesis of secondary metabolites (biosynthetic gene clusters—BGCs), which together comprise 8.6% of the genome. This percentage is approximately twice that of *Streptomyces* spp. genomes (which are a similar size to large myxobacterial genomes), suggesting that DK1622 has acquired additional BGCs by HGT [18].

### 1.2. Other Early Genome Sequences

After that of *M. xanthus* DK1622, the next complete myxobacterial genome to be published was that of *Sorangium cellulosum* So ce56 [21]. The *S. cellulosum* So ce56 genome sequence is 71.4 %GC, typical for myxobacteria, but 13.0 Mbp in length, nearly 4 Mbp larger even than DK1622. One-third of the *S. cellulosum* So ce56 genome is composed of paralogous genes, a lower proportion than for *M. xanthus* DK1622, but with a similar expansion of genes encoding enhancer-binding proteins (EBPs), two-component system (TCS) proteins and Ser/Thr protein kinases [21]. In both *S. cellulosum* So ce56 and *M. xanthus* DK1622, the expansion in number of these protein families is disproportionate to their genome size, with more such proteins per Mbp than any other sequenced bacterial genome at that time. Surprisingly, there was a complete lack of genome-wide synteny (conserved ordering of genes in a genome) observed between the genomes of *S. cellulosum* So ce56 and *M. xanthus* DK1622; however, individual genes exhibited a high degree of local synteny. A total of 1,474 of the 9,367 CDSs in the *S. cellulosum* So ce56 genome were found in syntenic clusters (mostly corresponding to operons), and the locally syntenic genes also exhibited particularly high sequence conservation with their *M. xanthus* DK1622 counterparts, implying conservation of both genome organisation and function of those genes [21].

Compared to that of *M. xanthus* DK1622, the *S. cellulosum* So ce56 genome has fewer protease and more carbohydrate metabolism genes plus additional genes for nitrogen assimilation [21]. This can be reconciled with *S. cellulosum* So ce56 being a prototroph which can grow on cellulose, while *M. xanthus* DK1622 requires amino acids for growth (as carbon and nitrogen sources), and is auxotrophic for (unable to synthesise) leucine, isoleucine, and valine [18,22].

Also published in 2007 was the draft genome sequence of *Plesiocystis pacifica* SIR-1, sequenced by The Gordon and Betty Moore Foundation Microbial Genome Sequencing project. SIR-1 is described as an aquatic organism with mesophilic salt tolerance and was isolated from a beach on the Japanese Island of Iriomote-jima. Despite being spread over 237 contigs, the genome sequence seems to be close to complete, spanning a total sequence length of 10.6 Mbp. Incomplete genome sequences can be characterised by their N50 and L50 values, where L50 is the minimum number of contigs (*x*) that together add up to half the total sequence length, and L50 is the length of the *x*^th^ largest contig. For *P. pacifica* SIR-1, the L50 value is 40 and N50 value is 82,268 bp which are typical values for large myxobacterial genomes. The complete genome of another marine myxobacterium, *Haliangium ochraceum* SMP-2, was published shortly after that of *P. pacifica* SIR-1 [23]. Although myxobacteria have traditionally been viewed as soil bacteria, an increasing number of marine examples have been described. SMP-2 was isolated from seaweed and grows optimally at 2% (*w*/*v*) NaCl. As is typical for myxobacteria, it is predatory and forms multicellular fruiting bodies, with a large (9.5 Mbp), high %GC (69.5%) genome [23].

After *H. ochraceum* SMP-2, the next myxobacterial genomes to be completely sequenced belonged to *Corallococcus coralloides* DSM 2259, *Stigmatella aurantiaca* DW4/3-1 and *Myxococcus fulvus* HW-1. All three are typical myxobacterial genome sequences, being large (9–10.3 Mbp), with a high %GC content (>67%), sharing synteny with each other and with *M. xanthus* DK1622 [24,25,26]. *M. fulvus* HW-1 (reclassified as *Myxococcus macrosporus* HW-1 in October 2018) is a halotolerant marine strain which forms fruiting bodies in low salinity conditions, but which can sporulate without fruiting in saltwater [26]. *C. coralloides* DSM 2259 produces fruiting bodies resembling coral, and it belongs to the most common myxobacterial genus isolated from soils alongside *Myxococcus* [27]. *S. aurantiaca* DW4/3-1 was first sequenced in draft form (released as 579 contigs in Sep 2006), before having its genome completely sequenced four years later [24]. Like *C. coralloides* DSM 2259, *S. aurantiaca* DW4/3-1 also produces morphologically complex fruiting bodies—in this case, with sporangioles mounted on a stalk. Comparisons with the genome sequences of other fruiting myxobacteria showed a lack of conservation of genes involved in fruiting across these myxobacteria, implying the genetic program underlying multicellular development is much more plastic than had been expected [24].

After the publication of the *A. dehalogenans* 2CP-C genome sequence in 2006 [17], genome sequences for a further three members of the genus were made public between 2007 and 2009, with one of those genomes (*Anaeromyxobacter* sp. Fw109-5) subsequently being described in the literature [28]. *Anaeromyxobacter* is an unusual myxobacterial genus as its members do not produce multicellular fruits and they have small genomes for myxobacteria—typically approximately 5 Mbp. *A. dehalogenans* 2CP-C is microaerobic and metabolically versatile, with various gene clusters for electron transport chain complexes acquired by HGT as well as by vertical descent from the ancestral myxobacterium [17]. *Anaeromyxobacter* sp. Fw109-5 is a metal-reducing strain, with the potential for application to the bioremediation of heavy metal-contaminated sites [28].

### 1.3. Expanding Coverage and Increasing Depth

**Figure 1:**
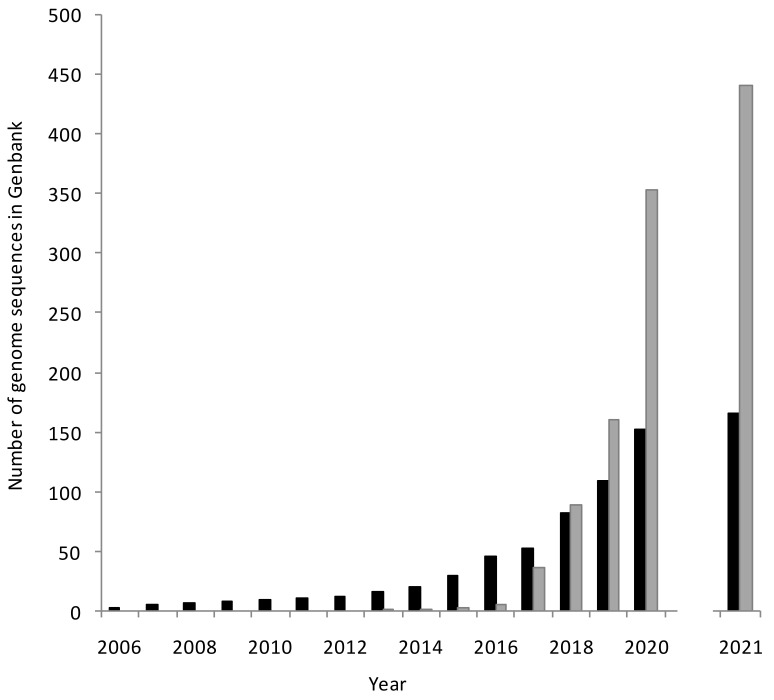
An exponential increase in myxobacterial genome sequencing. The numbers of genome sequences from cultured strains (black columns) and MAGs (grey columns) available at the end of each year are shown. The columns for 2021 only include genomes and MAGs published in the first six months of the year.

By 1st July 2012, two draft and ten complete myxobacterial genome sequences were publicly available, from eleven different organisms, spanning eight different genera [19]. Summary statistics of those 12 genome sequences are shown in Table 1. Since then, new sequencing technologies have reduced the cost and increased the accessibility of genome sequencing (see Segerman [29] for a recent review of developments in DNA sequencing technology). As of 2021, commercial services are typically offering 30x coverage of a bacterial genome for less than $70 USD, making genome sequencing an affordable activity even for undergraduate projects and laboratories without large grant incomes. This has resulted in a dramatic exponential increase in the number of available myxobacterial genome sequences (Figure 1).

**Figure 2:**
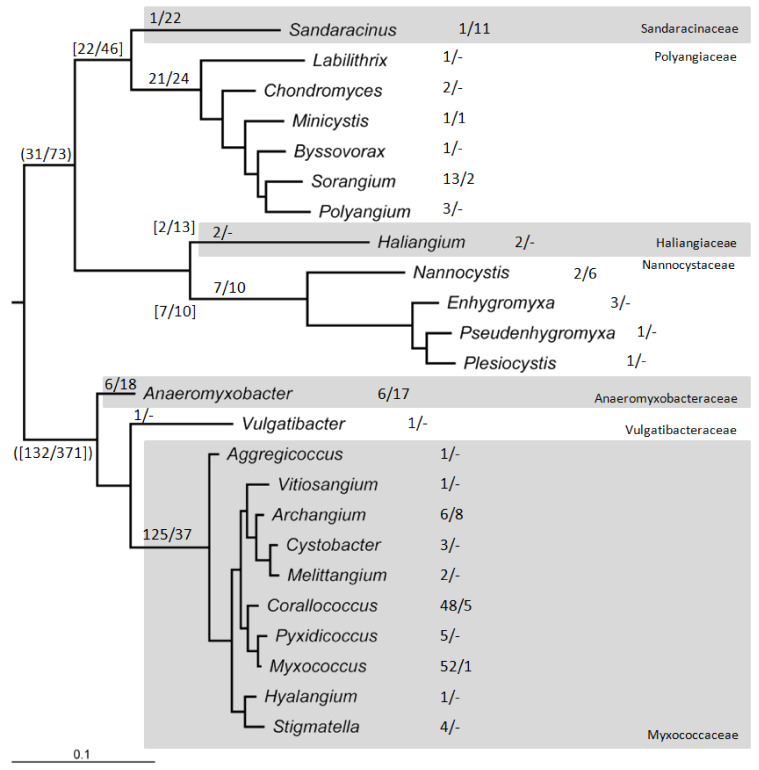
Phylogenetic tree showing the number of genome sequences and MAGs available for sequenced myxobacterial taxa. The tree was produced using 16S rRNA gene sequences from the type strain of each myxobacterial genus (Appendix A). Looking down the tree, families are alternately shaded grey and white. Numbers denote sequenced genomes/MAGs and are shown for each genus, family, order [in square brackets] and class (curved brackets). The Haliangiales and Nanncystales orders each comprise a single family (Haliangiaceae and Nannocystaceae, respectively), while the Myxococcia class contains a single class [Myxococcales]. Not all sequenced organisms/MAGs are taxonomically defined down to the genus, family or order levels (Supplementary Table S1).

It is now possible to routinely sequence newly isolated organisms from the environment, engineered strains, and evolved strains from evolution experiments [30,31,32]. The increase in genome sequencing activity has provided genome sequences for myxobacterial taxa previously lacking sequenced representatives, and provided more examples of sequenced individuals within key taxa, giving insights into genomic variation within those taxa and the typicality of laboratory model organisms. Figure 2 shows the number of genome sequences currently available for each myxobacterial taxon (as of the 1st July 2021), highlighting a relative dearth of sequences from within families *Vulgatibacteraceae*, *Haliangiaceae*, and *Sandaracinaceae*.

Supplementary Table S1 provides details of all 163 myxobacterial genome sequences deposited in GenBank as of the 1st July 2021, including details of the taxonomy of the organism, the size and %GC of its genome, the number of contigs, date of release and relevant accession numbers. Among the 163 available myxobacterial genomes are 24 draft genomes which we have sequenced and are describing here for the first time (Table 2). Eight of the 24 genomes are from previously undescribed strains, and for those, we also specify where the soil samples were taken which yielded each strain (all in West Wales, UK).

**Figure 3:**
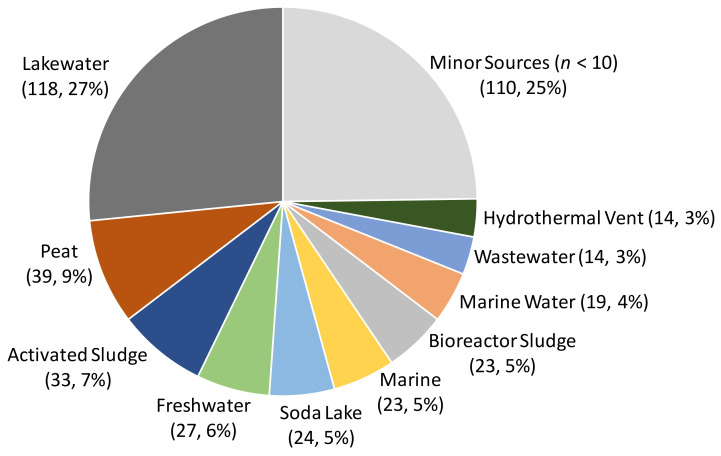
Sources of samples from which myxobacterial 444 MAGs have been derived. The ten sources which have yielded the largest numbers of MAGs are indicated.

The high coverage of sequencing possible in meta-genome sequencing has also allowed the reconstruction of genomes from uncultured myxobacteria (metagenome-assembled genomes—MAGs). For instance, the draft MAG of *Sandaracinus* sp. NAT8 was reconstructed from metagenomic sequence data generated from ocean samples [34]. The first myxobacterial MAG was added to Genbank in 2013 and within just five years the number of MAGs in Genbank exceeded that of genome sequences from individual strains (Figure 1). Despite their traditional description as soil bacteria, it remains surprising that approximately two-thirds of myxobacterial MAGs originated from aquatic samples—both saline and freshwater (Figure 3), while less than 20% originated from soil samples, although this may reflect bias in selection of sample sources for metagenomic studies rather than having any ecological significance. Supplementary Table S1 also provides details of all 444 MAGs deposited in GenBank as of the 1st July 2021, including the source of the sample which yielded each MAG.

**Figure 4:**
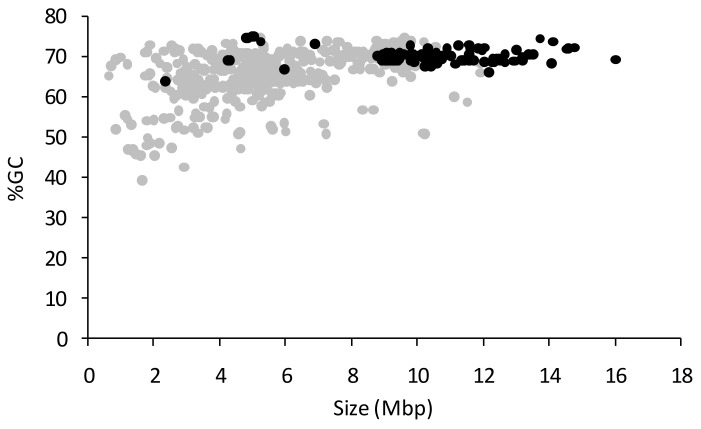
The relationship between genome size (Mbp) and %GC for myxobacterial genome sequences (black) and MAGs (grey).

Figure 4 shows the relationship between %GC and genome size for myxobacterial genomes and MAGs. Only one of the 163 myxobacterial genome sequences derived from pure strains has a %GC content below 66%, compared to 202 MAGs (46%). Similarly, while 93% of genome sequences from cultured strains have a size above 8.8 Mbp, only 12% of MAGs are that large. It therefore seems highly likely that a large proportion of the ‘myxobacterial’ MAGs in Genbank are not actually myxobacterial and should be treated with caution.

For the remainder of this paper, when we refer to genome sequences, we only consider those from cultured strains and do not include MAGs unless explicitly stated.

### 1.4. Genome Sequences and Myxobacterial Classification

In order to understand how genomes evolve as sister lineages diverge, forming new species, genera and families, we need to define the taxonomic relationships between genome-sequenced organisms. Currently, classification of novel myxobacterial taxa requires a polyphasic comparison with pre-existing taxa. Comparators include a variety of phenotypes/properties, typically including fruiting body morphology, colony morphology, cell morphology, nutritional requirements, DNA–DNA hybridisation, optimum growth conditions, fatty acid profiles and enzyme activities [35]. The ability to routinely PCR-amplify and DNA sequence the 16S rRNA gene of organisms led to the inclusion of 16S phylogenetic analysis as a requirement for classification and an objective tool for comparison of large numbers of strains (e.g., [36]). The phylogenetic approach allowed the facile assignment of environmental isolates to individual species. By convention, if the 16S gene sequence of an isolate shares >99% identity with that of the type strain for a species, it can safely be assumed to belong to that species.

Genome sequences are increasingly being used to support taxonomic assignment. DNA–DNA hybridisation (DDH) is an experimental approach, which assesses the sequence similarity of DNA from two sources by measuring the melting temperature of hybridised DNA, and has been used widely in taxonomy. DDH can be calculated directly from genome sequences (as digital DDH or dDDH values) and metrics for genome-wide sequence comparisons have been developed for inter-species and inter-genus comparisons [37]. The ANI (average nucleotide identity) assesses the percentage identity of all genes shared by two genomes, not just the 16S gene, and an ANI value below 95% is good evidence that two genome sequences come from different species [37]. ANI and dDDH-based approaches work equally well on draft and complete genomes. With genome sequences now available for most myxobacterial taxa, it is possible to robustly assign isolates to taxa and identify isolates which might represent novel taxa using their genome sequences alone.

For instance, environmental isolates CA053C, AB025A, AB025B, and AB036A have been described previously and assigned to a genus [33], their genome sequences have also been described [38], but their species membership has not been reported. To define their species, their genome sequences were compared to those of all *Corallococcus* spp. and *Myxococcus* spp. type strains and ANI values calculated. Each strain had an ANI value >95% when compared with only one type strain and no other, allowing assignment to that species (CA053C is *Corallococcus llansteffanensis*, AB025A and AB036A are *M. xanthus*, AB025B is *M. fulvus*). Similarly, Ahearne et al. [39] used ANI to show that *Archangium primigenium* ATCC 29037 is misclassified, actually belonging to a species of *Melittangium*. We have used this approach to identify the species of 37 genome-sequenced strains which were previously only assigned to a genus (indicated in Supplementary Table S1).

There are several advantages to sequence-based taxonomy compared to the polyphasic/16S approach, which can have limited resolution, with no clear criteria for delineating taxa and which can be subject to considerable experimental variation between laboratories [36,40]. In contrast, digital approaches are reproducible, objective, unambiguous and leverage the sequence of every conserved gene. Genome sequence-based approaches have typically supplemented traditional polyphasic approaches in myxobacteria rather than replacing them [41,42]; however, it seems likely that genome sequences will soon be a requirement for definition of a new taxon and may also soon be considered ‘type material’ [43]. At the moment, type material means cultures of an organism deposited at two international culture collections, thus only culturable organisms can be used to define new taxa. Allowing genome sequences as type material allows taxonomic assignment of uncultured taxa and genomes assembled from metagenomic datasets.

Above the level of the genus, genome sequence analysis has recently allowed a fundamental reclassification of the myxobacteria [44]. Prior to Waite et al.’s study [44], the myxobacteria were classified as order Myxococcales within the class Deltaproteobacteria, and contained three sub-orders (Cystobacterineae, Nannocysterineae and Sorangiineae), containing eight families (Myxococcaceae, Archangiaceae, Vulgatibacteraceae, Phaselicystidaceae, Polyangiaceae, Sandaracinaceae, Nannocystaceae, and Haliangiaceae), seven of which (all but Phaselicystideae) had at least one genome sequence available.

The Waite et al. genome sequence-based reclassification [44] elevated the myxobacteria to form their own phylum (Myxococcota), with two constituent classes (Myxococcia and Polyangia). Class Myxococcia contains a single order (Myxococcales), while class Polyangia contains three (Polyangiales, Nannocystales and Haliangiales). Seven families are distributed between the four orders—the one genus (*Phaselicystis*) within the Phaselicystideae is proposed to be subsumed into Polyangiaceae thereby rendering Phaselicystideae obselete. Archangiaceae is similarly rendered obselete by moving its constituent genera into family Myxococcaceae and a new family (Anaeromyxobacteraceae), which accommodates *Anaeromyxobacter* spp. [44]. Waite et al. [44] also suggest the existence of three further classes (containing four novel orders and five novel families) represented entirely by MAGs. The revised classification provide by Waite et al. [44] is that used in Supplementary Table S1 and Figure 2.

## 2. Myxobacterial Genome Biology

The availability of genome sequences for defined taxa means it is now possible to investigate how genome properties and gene presence/absence vary within and between taxa. Studies have focussed on gene families with large numbers of members in myxobacterial genomes, genes known to be involved in hallmark myxobacterial behaviours, and how the genome changes during evolution.

### 2.1. Pan-Genomics

Sequencing the genomes of multiple strains within a single bacterial species revealed that strains often have genes which are lacking in other members of the species. This led to the concept of a species ‘pan-genome’, which consists of a ‘core genome’ of genes present in all members of the species, and an ‘accessory genome’ containing genes which are present in the genomes of some but not all members [45]. ‘Core’ genes typically include essential/housekeeping genes and genes required for defining properties exhibited by that species, while ‘accessory’ genes are typically not essential but can confer additional properties on the strains which contain them. Wielgoss et al. [46] found that in *M. xanthus*, gene gain/loss from the accessory genome was faster than amino acid residue substitution rates in core genes by more than an order of magnitude.

Pan-genomes can be considered ‘open’ or ‘closed’ [47,48]. In species with closed pan-genomes, individual strains have very similar constituent genomes, entirely composed of core genes and with a very small number of accessory genes [48]. In contrast, members of species with open pan-genomes have relatively small core genomes with a larger proportion of each genome composed of accessory genes. Open pan-genomes continue to increase in size as more genome sequences are considered [47], which can be due to individual members acquiring novel genes by HGT or by lineage specific duplications and diversification.

**Figure 5:**
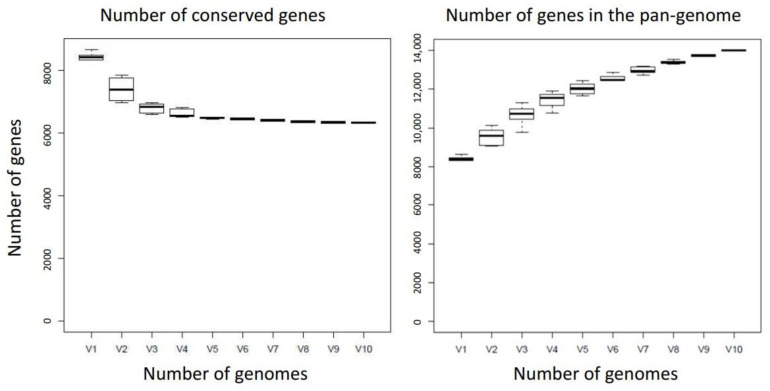
The *Corallococcus exiguus* pan-genome. The number of core genes (**left**) and total number of genes (**right**) for the pan-genome are shown as a function of the number of genomes included (V1–V10). Boxes show the median number of genes ± 1 standard deviation, whiskers show ± 2 standard deviations.

Figure 5 shows the pan-genome of ten randomly selected strains of *Corallococcus exiguus*, plotting the number of core genes and the total size of the pan-genome as a function of the number of genomes considered. Plots were generated using ROARY [49] and are very similar to those of *M. xanthus* [50]. The core genome falls rapidly from the mean genome size of 8400 genes to 6300 genes as more genomes are considered, showing that on average each strain’s genome is composed of 75% core genes and 25% accessory genes. The pan-genome size increases from 8400 genes as more genomes are added, indicating it is an open pan-genome, containing 14,000 genes after 10 genomes are included, i.e., more than half of the pan-genome is composed of accessory genes.

The pan-genome concept can be applied at any taxonomic level—for instance, the core genes of a genus would be those found in all species within that genus, while the accessory genes might be found in just some species within that genus. The pan-genomes of genera *Corallococcus* and *Myxococcus* have also been described [30,42]. At the genus level, the core genomes are much smaller than those of individual species (core genes comprising less than 30% of the average genome), and more than 60% of genes in the accessory genome are found in single species (singletons), indicating considerable variation in gene content between species within each genus [42]. There are also considerable differences between the pan-genomes, despite the constituent species being similarly diverse. The *Corallococcus* pan-genome had three times more core genes than the *Myxococcus* pan-genome which was larger and more open. In both genera BGCs were found to be enriched in the accessory genome, and were found in multiple strains at a much greater frequency than other accessory genome genes, implying they are preferentially conserved and provide a selective advantage [42].

### 2.2. Comparative Studies—Gene Repertoires

Having genome sequences available has allowed focussed investigations into the genetic repertoire of myxobacteria. Since the first myxobacterial genomes were sequenced, it has been clear that certain types of genes are unusually abundant in myxobacterial genomes, and several publications have now described those gene sets in detail.

BGCs are relatively abundant in myxobacteria and easily identified by sequence homology, with tens of BGCs in most myxobacterial genomes [51]. The metabolites synthesised by BGCs can be predicted by tools such as antiSMASH [52], which reveal a relative abundance of BGCs which produce terpenes, bacteriocins, polyketides and non-ribosomal peptides [51,53]. Several BGCs are associated with cytochrome P450s, which are also abundant in myxobacterial genomes [54]. Most BGCs are thought have been acquired relatively recently by HGT and are part of the accessory genome [18,30,42]. Due to the diversity of their BGCs, it is thought that myxobacteria represent an untapped reservoir of undiscovered natural products, accessible through genome-mining [55,56,57].

Myxobacterial genomes also contain a very large number of Ser/Thr kinases, which are important components in phosphorylation-dependent signalling pathways, predominantly found in eukaryotes [58]. Despite being generally uncommon in bacteria, individual myxobacterial genomes can encode more than a hundred Ser/Thr kinases, often with unusual combinations of domains for signal transduction (including protein-protein interaction domains, ligand-binding domains and additional kinase/phosphatase domains). The large numbers of kinases are disproportionate with genome size as a consequence of extensive gene duplication [58]. As might be expected, the number of genes encoding Ser/Thr phospho-protein phosphatases in myxobacterial genomes correlates with their number of Ser/Thr kinases, but in far smaller numbers [59].

TCS signalling pathways are the dominant signalling pathways in bacteria and are comprised of a sensor kinase protein which autophosphorylates upon stimulation and then transfers the phosphoryl group to a partner response regulator (RR). Large myxobacterial genomes typically contain more than 250 TCS genes, which is far more than would be expected even for their large genome sizes [60,61]. Myxobacterial TCS genes are often found in complex clusters of TCS genes, or encode TCS proteins with multiple TCS domains, implying a large degree of signal integration between TCS pathways. RRs usually have effector domains in addition to their phosphorylatable ‘receiver’ domain, but a large proportion of myxobacterial RRs lack such domains, implying they regulate downstream processes via protein-protein interactions [60].

CheY is an example of such a receiver-only RR, which receives phosphoryl groups from the kinase CheA in the TCS signalling pathway governing chemotaxis in diverse organisms. Myxobacterial motility is mechanistically complicated [6], with two distinct engines giving rise to two modes of motility—single-celled ‘adventurous’ motion (A-motility), or communal ‘social’ movement (S-motility). Myxobacterial genomes encode multiple CheA-CheY ‘chemosensory’ systems (*M. xanthus* DK1622 has eight), some of which are involved in regulating motility, while others regulate diverse behaviours, including fruiting body development. Some chemosensory systems are conserved broadly across the myxobacteria, while others seem to have been acquired by relatively recent HGT [62].

Prior to the advent of myxobacterial genome sequencing, numerous studies harnessed the power of bacterial genetics to identify large numbers of genes involved with development and/or motility [63]. Having genome sequences then enabled studies into the conservation and universality of those genes within the myxobacteria. For instance, Huntley et al. [24] showed there was substantial commonality between the developmental programs of fruiting myxobacteria, although substantial plasticity in the program was observed when comparing distantly related myxobacteria [19]. Whitworth and Zwarycz [64] found that genes encoding signalling proteins were enriched in the core genome (with virtually all TCS genes being core), and that within the developmental network, plasticity could be observed even within closely related strains.

### 2.3. Genome Organisation

In addition to the presence/absence of genes in a genome, their relative location and position-dependent properties are also important considerations. For instance, genes of related function are often grouped together into operons under the control of a shared promoter. During DNA replication, genes tend to maintain their relative order on the genome, a property called synteny. However, recombination events, deletion of genes and the incorporation of new genes from duplications or HGT can change the relative order of genes in a genome [65].

Huntley et al. [19] assessed ‘macro’-synteny across myxobacterial genomes by creating dotplots which mapped the positions of homologues for a pair of genomes. Closely related myxobacterial genomes exhibit a pronounced diagonal line due to synteny (e.g., *M. macrosporus* HW-1 compared with *C. coralloides* DSM 2259). However, some genome comparisons (e.g., comparing *M. xanthus* DK1622 with *S. aurantiaca* DW4/3-1) give X-patterns, which are likely due to symmetric interreplichore inversions. Such inversions are the result of recombination between DNA at replication forks, which proceed bi-directionally around the circular chromosome from the *oriC* origin of replication to the *ter* terminus [66]. Comparing more distantly related myxobacteria (e.g., *H. ochraceum* SMP-2 compared with *M. xanthus*), gives dotplots which lack any obvious macro-syntenic relationships [19].

Micro-synteny was observed by Pérez et al. [58] in their investigation into the myxobacterial kinome. Genes encoding Ser/Thr kinases often had conserved local context, with neighbouring genes also being found alongside orthologues in other genomes. Ser/Thr kinase genes have been extensively duplicated in some myxobacterial lineages, and the resulting paralogues are often found close to one another in the genome [58]. Similar patterns of local duplication and micro-synteny are also seen for TCS genes [61].

Another common feature of genome organisation is an asymmetric nucleotide composition of the two strands of DNA. This asymmetry is known as GC skew and it inverts along a DNA strand as the strand changes between being the leading or lagging strand [67]. Changes in asymmetry along a chromosome can therefore be exploited to identify the position of the origin *oriC* gene (usually next to the *dnaA* gene) and the terminus *ter* genes. Notably, the *S. cellulosum* So ce56 genome does not display the usual inversion of GC skew, precluding its use to identify *oriC* [21]. However, using a more complex algorithm, it was subsequently suggested that the *oriC* gene was located next to *dnaN*, nearly 2 Mbp from the *dnaA* gene [68].

Some genes and genome properties are not evenly distributed across myxobacterial genomes. For instance, the *S. cellulosum* So ce56 genome sequence has a region between 8.5 Mbp and 12.5 Mbp, spanning the origin, which is enriched for insertion sequences and contains 90% of predicted genomic islands [21]. In *M. xanthus* DK1622, three clustered interspaced short palindromic repeats (CRISPRs) are found clustered close to the origin, while the four rRNA operons seem to occur in two pairs, with members of each pair approximately the same distance from the origin but opposing each other on the chromosome [69]. A non-random distribution of development genes has also been described, with developmental genes involved in intra-cellular signalling being enriched around the origin compared to inter-cellular signalling genes [70]. With all of these observations, it will be interesting to investigate whether such apparently non-random distributions are due to selective pressures based on the functional roles of the genes, whether genomic location affects gene expression/dosage, or whether genomic location is merely a random consequence of evolutionary mechanisms and heritage.

A particularly variable region in the *M. xanthus* genome was found by Wielgoss et al. [46] when they investigated the whole genome sequences of 22 strains exhibiting colony-merger incompatibilities. Compatibility type dictates whether two expanding colonies are able to merge together during growth and the genome sequences of strains from 11 compatibility types revealed four regions which had a high density of SNPs (single nucleotide polymorphisms). For one of the four regions, spanning 150 kbp, the pattern of SNPs matched compatibility type groupings, as did the presence/absence of genes in the region. The region contained prophages (integrated temperate bacteriophages) and several potential toxin genes, prompting suggestions the region dictates compatibility type [46].

A final aspect of genome organisation considered briefly here is the distribution of gene between replicons. While some bacteria contain two chromosomes, all myxobacteria contain single chromosomes and were thought not to harbour plasmids until recently. Plasmids could be introduced into *M. xanthus* but could not be maintained without integrating into the chromosome by homologous recombination, or by integration into a temperate phage *attB* locus [71,72]. The first autonomously replicating myxobacterial plasmid, pMF1, was discovered in *M. fulvus* strain 124B02 [73]. Maintenance of the plasmid is through a toxin–antitoxin system, constituted by a toxic DNA nuclease and a co-transcribed immunity protein [74]. The plasmid contains 23 predicted CDSs; two encode the toxin–antitoxin system, seven are part of the replication and partitioning systems and the remainder have no obvious function, but homologues can be found occasionally in other myxobacterial genomes. The complete genome sequence of *M. fulvus* 124B02 also revealed the presence of paralogues of the pMF1 toxin–antitoxin gene pair in the strain’s chromosome [75].

### 2.4. Genome Evolution

A large number of studies have used contemporary myxobacterial genome sequences to make inferences regarding the evolutionary processes acting on myxobacteria and their genomes. As would be expected, the types of mutation described in myxobacteria are generally seen across the bacteria. For instance, evidence for recombination between replichores, local gene duplications, gene gain/loss, point mutations, small and large insertions/deletions, and gene fusion/fission can all be observed even when just considering the TCS genes of myxobacteria [76].

There is a metabolic burden associated with the replication of genes, and for bacteria which compete by outgrowing one another, there is consequently a selective pressure to reduce their genome sizes by purifying selection [77]. Genes arriving by HGT will mostly be detrimental to the host cell, and liable to be lost quickly unless they confer a selective advantage [78]. For typical bacteria like *Escherichia coli*, with relatively occasional HGT and small genomes, an adaptationist view of bacterial genomes would suggest that the majority of genes in the genome confer a selective advantage and have a functional role. The failure to observe a phenotype upon deleting a gene could be simply explained if the gene had a niche-specific function which could not manifest under laboratory conditions. For organisms with open pan-genomes, genome size will presumably be a consequence of the relative rates of gene gain and gene loss, and therefore genomes with weak streamlining selection would be expected to be larger.

Myxobacteria are slow-growing organisms and seem to out-compete neighbouring organisms by preying upon them rather than outgrowing them. Huntley et al. [19] discuss the trade-off between translational yield and translational robustness, suggesting that myxobacterial evolutionary success is due to metabolic ‘efficiency’ rather than speed. Presumably, slow but efficient growth results in a weakened pressure to streamline, which coupled with high rates of HGT results in the large size of myxobacterial genomes. (For this reason it would be interesting to determine whether *Anaeromyxobacter* spp. are predatory, given their small genome sizes.) There is a link between slow growth, large genomes and %GC (a GC base pair is more metabolically expensive to produce than an AT base pair), but any causality is yet to be proven [79]. Nevertheless, it is intriguing that although taxonomically distant from the myxobacteria, the slow-growing, high %GC, BGC-replete members of genus *Streptomyces* have recently been shown to have widespread predatory activity too [80].

A lack of streamlining selection would allow accessory genomes to accumulate genes which do not necessarily confer an immediate fitness advantage, but are instead contingent—for instance, potentially providing a future selective advantage. In the context of multicellular development, reduced streamlining may provide more time for strains to assimilate newly acquired regulators into the developmental program before they are lost from the genome, increasing plasticity and the potential for regulatory innovation [64]. In the context of predation, this may allow maintenance of a diverse arsenal of potentially useful weapons—a sensible strategy considering the inevitability of resistance evolution in prey organisms, and which chimes with the broad prey range exhibited by myxobacterial predators [38].

Nair et al. [81] investigated genome changes in co-evolving co-cultures of *M. xanthus* and *E. coli*. They found reciprocal adaptation between the predator and prey, stimulation of mutation rates and the emergence of mutator genotypes. It would seem that despite taking a generalist approach to predation, myxobacteria can also evolve to increase their predation of particular prey, and that predation per se can drive innovation. Predation could also stimulate innovation through HGT of genes into predator genomes from DNA released by their lysed prey, although genomic signatures of such events are elusive [18].

Nevertheless, HGT from non-myxobacteria would seem to be a major driver for the evolution of myxobacterial accessory genomes: most genes in the accessory genomes of myxobacterial species are singletons (i.e., found only in single genomes), and little exchange is observed between myxobacteria, except between closely related strains [38,46]. Rates of gene gain and loss are high relative to the rate of speciation, yet sequence-based evidence for HGT (e.g., regions with anomalous GC skew or %GC), is missing from myxobacterial genomes [18,19]. Either newly acquired genes are converted to resemble the host genome very quickly (a process called amelioration), or there is selection such that only ‘myxobacterial-like’ sections of DNA are effectively retained/integrated.

Myxobacteria can take up foreign DNA by transformation and transduction, but conjugation has not been observed. *M. xanthus* is naturally competent and has been shown to acquire drug-resistance genes from other bacteria [82,83]. Relevant to transduction, several temperate bacteriophages of *Myxococcus* spp. have been identified, and various strains of *M. xanthus* carry prophages of *Mx alpha* in their genomes [84]. The prophages reside within the variable region identified by Wielgoss et al. [46] that is responsible for colony merger compatibility and they contain toxin/antitoxin systems responsible for kin discrimination [85]. The incorporation of viral and other incoming DNA into the myxobacterial genome is likely to depend upon the activity of CRISPR-Cas systems, and in *M. xanthus* DK1622 two of the three CRISPR-Cas systems are involved in another social phenomenon—multicellular development [84]. In the original Genbank annotation of the DK1622 genome, 27 CDSs spread over eight loci were annotated as phage proteins, including six recombinases (integrases/excisionases). The *M. xanthus* DK1622 genome also encodes 53 transposases, belonging to seven different IS (insertion sequence) families, suggesting that myxobacterial genomes are shaped by the frequent passage of mobile genetic elements.

### 2.5. Comparative Studies—Evolution of Specific Myxobacterial Systems

Many studies have investigated the evolution of particular myxobacterial genes and behaviours by comparative analysis of extant genes. The examples below are illustrative rather than comprehensive, but give an idea of the breadth of research activity.

Goldman et al. [86] investigated the evolution of fruiting body formation, finding that three-quarters of developmental genes were inherited vertically. Looking across the *M. xanthus* DK1622 genome, they also identified frequent acquisition of metabolic genes by HGT, including several components of the electron transport chain, reminiscent of the observations of Thomas et al. [17] for *A. dehalogenans* 2CP-C. Other examples of myxobacterial genes gained by HGT include those encoding sterol biosynthesis, an unusual phenomenon in bacteria, which myxobacteria likely acquired from eukaryotes [87].

Other studies have investigated the origin of genes which appear to have arisen de novo within myxobacterial evolution. The Pxr non-coding RNA which regulates fruiting body formation seems to have evolved within the Cystobacterineae sub-order (now order Myxococcales), while the devI regulator of fruiting seems to be a very recent innovation within *M. xanthus* [88,89]. Sequence analysis of 120 strains isolated from six fruiting bodies has shown that genomic changes are concentrated in ‘selection hot-spots’ and also characterised the rate of endemic diversification [32].

Luciano et al. [90] used a phylogenomic approach to characterise the evolution of candidate genes potentially involved in gliding motility. Using evolutionary and synteny-based arguments they identified three genetic clusters encoding basal motility machinery. Their results also suggested a model for the evolution of gliding motility wherein a core set of ancestral genes of unknown function subsequently recruited extra functional modules [90]. A similar mode of evolution has also been suggested for the type IV pili-based motility systems of myxobacteria [91].

It is also worth noting here an intriguing hypothesis concerning myxobacterial evolution, which suggests that an ancestral myxobacterium may have evolved into a non-myxobacterium. The syntrophy hypothesis proposes that the eukaryotic common ancestor was the result of a tripartite symbiosis involving a myxobacterium-like deltaproteobacterium, which became the eukaryotic cytoplasm [92]. The hypothesis suggests the involvement of a myxobacterial-like organism due to many features of myxobacterial biology which are unusual for bacteria, but common to eukaryotes, including (among many examples) defensins, eukaryotic-like Ser/Thr kinases and enhanceosomes [58,93,94].

## 3. Myxobacterial Post-Genomics

The availability of a genome sequence is a pre-requisite for several ‘omics technologies, particularly transcriptome and proteome analyses. The widespread application of such approaches to myxobacteria has led to the generation of large numbers of ‘omics datasets, albeit mainly for *M. xanthus*. Increasingly, ‘omics studies and other post-genomic approaches are providing holistic insights into myxobacterial taxonomy, evolution and molecular biology.

### 3.1. Molecular Genetics

The availability of a genome sequence can inform us about the function and origin of its constituent genes via comparative genomics analyses and it allows the directed study of individual genes or sets of gene in that genome (e.g., [95,96,97]. The roles of genes can be inferred if they share homology with genes of known function in other organisms, but comparative genomics also makes possible the identification of candidate genes with no obvious functional relationship with the role, including those encoding hypothetical proteins [98]. For example, Luciano et al. [90] made functional predictions of gliding motility genes using synteny-based arguments, while Sutton et al. [38] correlated gene presence/absence with predatory activity to identify candidate predation genes. Genome re-sequencing of spontaneous mutants or members of mutant libraries also allows the identification of genes responsible for the phenotypes under study, a strategy exemplified by the identification of the Pxr non-coding RNA [99].

There is a well-established genetic toolbox for *M. xanthus* that can be used to investigate gene function experimentally, reviewed by Murphy and Garza [9]. Until recently, plasmids that could replicate in myxobacteria were unknown, but plasmids could be introduced into myxobacteria as suicide vectors. Integration into the chromosome could be engineered to happen by homologous recombination with PCR-cloned DNA, or by inclusion of a phage integrase gene and attachment site to direct insertion into the chromosomal *attB* site (e.g., [100,101]. Single recombination of a plasmid containing an internal fragment of a gene could result in insertional disruption of the target gene, while the use of counter selectable makers such as *galK*, allowed the creation of in-frame unmarked deletions via double recombination (e.g., [102]). Since Murphy and Garza’s review, the genetic toolbox for myxobacteria has expanded significantly. A replicative plasmid was discovered in *M. fulvus* 124B02, which could be maintained in *M. xanthus* [73]. Inducible promoter systems have been developed, with induction by IPTG (isopropyl-β-D-thiogalactopyranoside), copper or vanillate [103,104]. More recently, genome editing systems have been developed for *M. xanthus*, which would not have been possible without the availability of its genome sequence. A Cre-Lox recombination system was used to engineer a 244 Kbp deletion in DK1622 [105], while CRISPR/Cas-based systems have been used to delete large BGC fragments and to stimulate the expression of a BGC heterologously expressed in *M. xanthus* [106,107].

### 3.2. Transcriptomics

Prior to the advent of genome sequencing, transcription was normally assayed in *M. xanthus* using northern blots or reporter genes. Typically, a promoterless *lacZ* gene would be cloned downstream of a promoter and introduced into the chromosome (typically at the *attB* site), with production of LacZ (measured by colorimetric assays) indicating transcriptional activity (e.g., [108]). Alternatively, a promoterless *lacZ* within the transposon Tn*5* *lac* allowed transcription to be assessed wherever a transposon had inserted into the chromosome [109]. Reverse transcriptase PCR (RT-PCR) increased the ease with which transcriptional assays of sequenced genes could be performed [110], and the arrival of the *M. xanthus* genome sequence allowed the approach to be applied to any gene in the chromosome.

Having the genome sequence of *M. xanthus* DK1622 also allowed the entire transcriptome to be assessed simultaneously, using microarrays initially, and then RNA-seq. The first microarrays were used to study EBPs (enhancer-binding proteins), which are a family of regulatory proteins which stimulate transcription from Sigma54-dependent promoters [95]. Fragments of 371 putative CDSs in the M1 genome sequence were amplified by PCR and spotted onto glass slides. In a two-colour experiment, RNA is extracted from cells grown in (e.g.,) two different conditions, at different time-points during development or from different mutant backgrounds. The RNA samples are reverse-transcribed into cDNA and the two samples of cDNA labelled with different fluorescent dyes. The labelled cDNA samples are then mixed and hybridised to DNA spots on the microarray slide. Up- or down-regulation of CDSs is observed based on the relative intensity of the two fluorescent dyes at each spot. Jakobsen et al. [95], used this approach to investigate changes in transcription during development, identifying 11 transcriptional regulators and six EBPs which were induced 12 h into fruiting body formation.

The first ‘genome-wide’ microarrays were developed by the *Myxococcus* Microarray Consortium and included spots for 88% of the CDSs in the *M. xanthus* DK1622 genome. Several studies used the arrays to identify which genes are regulated by transcriptional regulators, by comparing gene expression profiles of wild-type strains with those of strains carrying a mutation in the transcriptional regulator gene. For example, Diodati et al. [111], used the arrays to investigate the function of Nla18, a developmental EBP, by comparing transcription in wild-type cells with that of an *nla18* mutant. Surprisingly, in addition to developmental genes, >700 genes were differentially expressed during vegetative growth in the *nla18* mutant compared to the wild-type. Other regulators studied in this way included the response regulators DigR and PhoP4 and the non-coding RNA Pxr [112,113,114]. Bode et al. [115] used a similar approach to investigate the synthesis of isovaleryl-CoA by assessing transcriptional changes in *bkd* mutants, which are unable to synthesis isovaleryl-CoA via the branched-chain keto acid dehydrogenase complex. Genes identified as being up-regulated in *bkd* mutants included genes encoding an alternative pathway of isovaleryl-CoA synthesis (from 3-hydroxy-3-methylglutaryl-CoA).

Other studies used the microarrays to assess global patterns of gene expression changes associated with a particular biological process in wild-type strains. Shi et al. [116] investigated two-component system genes encoded in the *M. xanthus* DK1622 genome, and assessed which were differentially expressed during fruiting body formation. To help disentangle the regulation of sporulation from that of fruiting body formation, Müller et al. [117] undertook transcriptome profiling of glycerol-induced sporulation, which occurs in single cells without the normal requirement for fruiting body formation. The analysis identified an operon of eight genes up-regulated during sporulation which upon deletion were found to be required for sporulation, but did not affect fruiting body formation. Furusawa et al. [118] investigated differences in expression profiles between yellow and tan phase variants of *M. xanthus*, identifying 41 genes which were specifically up-regulated in yellow or tan variants, including a gene encoding a transcriptional regulator (HTH-Xre) which was subsequently shown to regulate phase switching.

RNA-seq is a method of transcriptome profiling which directly sequences the cDNA generated from a sample of RNA and maps cDNA reads to CDSs in the genome sequence, with the coverage of reads correlating with relative transcript abundance. It has largely superseded microarray profiling because it sequences all RNAs (including those transcribed from unannotated CDSs and non-coding RNAs), it provides quantitative data for each sample rather than a comparison between samples, and avoids problems associated with hybridisation and probe design [119]. Zhu et al. [120] used a transposon-based system to integrate a heterologous BGC (for the production of epothilones) into the genome of *M. xanthus*, and strains in which the BGC inserted at different genomic sites were found to produce different quantities of epothilones. To investigate those changes in expression, RNA-seq was used to provide a transcription profile of the entire genome in different insertion strains, and it was discovered that insertion of the BGC at different sites caused selective changes in transcriptional activity across the host genome.

Livingstone et al. [121] used RNA-seq to investigate transcriptome changes during *M. xanthus* predation of *E. coli*. Surprisingly, the presence of live prey significantly induced expression of just 12 genes, despite dead prey inducing expression of >1300 genes. This suggested that myxobacteria do not respond to prey presence per se, instead responding to the nutrients released when prey cells are killed. The RNA-seq approach allowed simultaneous investigation of prey gene expression, revealing the induction of >1500 genes in the prey upon exposure to the predator. Subsequent analysis of the RNA-seq data was also able to identify tens of non-coding RNAs in the *M. xanthus* transcriptome, many of which were differentially regulated by nutrient availability [69]. Subsequently, RNA-seq studies have investigated the regulation of fruiting body formation—variously identifying eight or ten distinct sets of expression profiles [122,123], differentiation of peripheral rods—specialised developmental cell types [124], and genes whose expression was induced by UV light—which included BGCs as well as genes of the LexA/SOS response [125].

### 3.3. Proteomics

In typical proteomic workflows, proteins are digested with trypsin, the resulting peptides are separated (e.g., by high-performance liquid chromatography) and their mass and ‘fragmentation fingerprint’ accurately determined using mass spectrometry (MS). This allows the sequence of the peptide to be deduced, which is then matched against a theoretical translation of the CDSs in the relevant genome, identifying and quantifying the protein from which the peptide was derived. In myxobacterial research these approaches were initially applied to proteins which had been separated by polyacrylamide gel electrophoresis—either as bands from one-dimensional gels, or spots from two-dimensional gels. For convenience, an entire lane of a one-dimensional gel could be divided into chunks for analysis, giving a semi-quantitative and low-resolution overview of a whole proteome. This approach was used to characterise the proteomes of the *M. xanthus* inner membrane, outer membrane, outer membrane vesicles (OMVs), and extracellular matrix [126,127,128,129].

A similar approach involves ‘mapping’ a proteome, using two-dimensional gel electrophoresis to separate proteins into discrete spots, and then identifying the proteins within each spot. Comparisons between proteomes can then be undertaken by identifying spots with changes in relative intensity, or by labelling two proteomes with different fluorescent dyes and then mixing them before running them on a single two-dimensional gel. Proteins which were relatively more/less abundant in one of the two proteomes would be highlighted in the map because of their coloration. Dahl et al. [130] used such an approach to investigate the spore proteins of *M. xanthus* and identified three previously unknown sporulation proteins (MspA, MspB and MspC). Spores produced by strains with mutations in the *mspA*, *mspB* and *mspC* genes had an altered cortex layer and were more sensitive to environmental stresses. Chao et al. [131] generated a proteome map for *A. dehalogenans* which included 559 proteins, and used the map to investigate the metabolic shift from growth on fumarate to growth on ferric citrate, which was found to affect the relative abundance of 239 proteins. To investigate the role of the transcriptional regulator ROK, Izzat et al. [132] also used two-dimensional fluorescence difference in-gel electrophoresis to compare the proteomes of wild-type and *rok* mutant strains, identifying 130 proteins which were affected by the *rok* mutation.

Gel-free systems are being increasingly used for proteomics, avoiding problems with labelling, quantification and gel loading. Hot on the heels of the *M. xanthus* and *S. cellulosum* genome sequences becoming available, their proteomes were characterised using gel-free approaches, identifying 631 and 952 proteins, respectively [133,134]. Several proteome studies using gel-free and gel-based approaches have focused on OMVs and other proteomes that have reduced complexity compared to cellular proteomes. Berleman et al. [135] and Whitworth et al. [136] investigated the OMVs of *M. xanthus* DZ2 and *M. xanthus* DK1622, respectively, while Zwarycz et al. [50] assessed proteome variation between the OMVs produced by ten independently isolated strains of *M. xanthus*. Whitworth et al. [136] also characterised the soluble secreted proteins and cytoplasmic proteins of *M. xanthus* DK1622 and found that the composition of the soluble supernatant proteome correlated significantly with that of OMVs, implying that lysis of OMVs may in large part dictate the composition of the soluble secreted proteome.

### 3.4. Metabolomics and Interactomics

While not relying directly on genomic sequence data, metabolomics studies can be enriched by genome sequences. For instance, Bolten et al. [137] cultivated cells of *S. cellulosum* So ce56 on ^13^C-labelled glucose and identified the metabolites which incorporated the ^13^C label using GC/MS (gas chromatography coupled with MS). The authors used the *S. cellulosum* So ce56 genome sequence to construct a model of its metabolic network. This allowed a system-wide inference of metabolic fluxes through the pathways of primary metabolism, identifying glycolysis and the pentose phosphate as the major catabolic pathways, with approximately equal fluxes through both. It was also found that the Entner–Douderoff and glyoxylate pathways were inactive in *S. cellulosum* So ce56, and that 90% of the ATP generated by the TCA cycle was consumed during cell maintenance rather than cell growth [137].

An interactomics approach based around high-throughput DNA sequencing is chromatin immunoprecipitation followed by DNA sequencing (ChIP-seq), in which a DNA-binding protein is cross-linked to its bound DNA, and an antibody used to immunoprecipitate the target protein. Bound DNA is released from the precipitate and sequenced, to reveal which parts of the genome are targeted by the DNA-binding protein. Robinson et al. [138] successfully used this approach on *M. xanthus* to identify 1608 putative binding sites for the developmental regulator MrpC, highlighting its involvement in multiple aspects of the developmental program. Sequence similarities between the 1608 putative binding sites allowed identification of a consensus sequence, which was shown to bind a form of MrpC in vitro.

## 4. Perspectives

In the 15 years following the publication of the first myxobacterial genome sequences, there has been an explosion in the number of myxobacterial genomes which have been sequenced. This has enabled comparative genomic analyses of diverse aspects of myxobacterial biology and has also made possible the application of post-genomic approaches for systems-level analyses of model myxobacteria. The resulting deluge of data has already provided holistic information about the molecular basis of model myxobacterial behaviours, and many more insights are surely yet to be gleaned from those datasets. Genome sequences and post-genomic datasets have generated numerous hypotheses, which can now be tested using molecular genetics approaches.

While model organisms are invaluable tools for investigating molecular genetics, myxobacterial genomes are highly variable and it is not clear to what extent model myxobacteria represent other members of their taxa. Comparative genomics and identification of homologous genes allow the transfer of knowledge between organisms, but we also need to investigate the functional or evolutionary significance of variations between members of the same taxon. For some myxobacterial taxa, we have tens of sequenced genomes, for other taxa we still have none.

As technology advances, what is currently science fiction can quickly become science fact and as costs decrease, advanced technologies become routinely accessible for greater numbers of scientists. In the near future, we would predict current major challenges in myxobacterial research to be overcome. Perhaps:

- Single-cell transcriptomics will be combined with advanced imaging techniques and single-cell tracking to investigate the epigenetic effects of life history on individuals in a population.
- MAGs will direct efforts to define and cultivate novel taxa which are currently unculturable.
- Genome editing and/or recombineering will be used to produce high-throughput combinatorial gene deletions for investigations into gene function.
- Single amplified genomes will provide insights into evolutionary processes within natural populations.
- Proteomics methods will be used holistically to assess post-translational modifications, particularly those associated with epigenetic regulation of metabolism and signalling.
- Artificial intelligence will be used to integrate multi-omic data and physiological data into systems models and to generate hypotheses for testing.

## Figures and Tables

**Table 1:** The first 12 published myxobacterial genome sequences (as reported by Huntley et al. [19]), ordered by release date. Sequences without a reference were published variously by: * The Institute for Genomic Research, ^†^ The Gordon and Betty Moore Foundation Microbial Genome Sequencing project, or ^‡^ the United States Department of Energy Joint Genome Institute.

Organism	Mbp	%GC	Contigs	Released	Source	Accession
*Anaeromyxobacter dehalogenans* 2CP-C	5.0	74.9	1	Jan 2006	[17]	GCA_000013385.1
*Myxococcus xanthus* DK1622	9.1	68.9	1	Jun 2006	[18]	GCA_000012685.1
*Stigmatella aurantiaca* DW4/3-1	10.3	67.4	579	Sep 2006	TIGR *	GCA_000168055.1
*Plesiocystis pacifica* SIR-1^T^	10.6	70.7	237	Jun 2007	G&BMF MGSP ^†^	GCA_000170895.1
*Anaeromyxobacter* sp. Fw109-5	5.3	73.5	1	Jul 2007	[28]	GCA_000017505.1
*Sorangium cellulosum* So ce56	13.0	71.4	1	Nov 2007	[21]	GCA_000067165.1
*Anaeromyxobacter* sp. K	5.1	74.8	1	Aug 2008	US DOE JGI ^‡^	GCA_000020805.1
*Anaeromyxobacter dehalogenans* 2CP-1^T^	5.0	74.7	1	Jan 2009	US DOE JGI ^‡^	GCA_000022145.1
*Haliangium ochraceum* SMP-2^T^	9.5	69.5	1	Oct 2009	[23]	GCA_000024805.1
*Stigmatella aurantiaca* DW4/3-1	10.3	67.5	1	Oct 2010	[24]	GCA_000165485.1
*Myxococcus macrosporus* HW-1	9.0	70.6	1	Jun 2011	[26]	GCA_000219105.1
*Corallococcus coralloides* DSM 2259^T^	10.1	69.9	1	Mar 2012	[25]	GCA_000255295.1

**Table 2:** Summary statistics of 24 genome sequences described here for the first time. The strains are environmental isolates and were assigned to species using genome-based taxonomic principles as described in the text (ANI and dDDH comparisons). Some of the strains (but not their genomes) have been described previously [33].

Strain	Taxonomy	Size (Mbp)	%GC	Contigs	CDS	L50	N50	Coverage	Genbank Accession	Area of Sampling	Reference
CA046B	*Corallococcus carmarthensis*	10.74	69.8	1079	8532	132	23389	71x	JABFJX000000000	Carmarthen	[33]
CA044C	*Corallococcus coralloides*	10.05	70	1379	8124	91	32261	65x	JABFJY000000000	Carmarthen	[33]
AB043B	*Corallococcus exercitus*	10.26	70.2	690	8102	119	26793	77x	JABFJV000000000	Capel Bangor	[33]
CA046A	*Corallococcus exercitus*	9.9	70.5	874	7710	146	21172	77x	JABFJW000000000	Carmarthen	[33]
AB032A	*Corallococcus exiguus*	10.44	69.6	972	8216	132	23729	60x	JABJTS000000000	Penglais Woods, Aberystwyth	[33]
AB038A	*Corallococcus exiguus*	10.57	69.4	631	8281	92	33924	72x	JABJTT000000000	Penglais Woods, Aberystwyth	[33]
AB039A	*Corallococcus exiguus*	10.54	69.4	716	8269	111	28467	91x	JABJTU000000000	Penglais Woods, Aberystwyth	[33]
AM006	*Corallococcus exiguus*	10.59	69.5	865	8306	108	29172	67x	JABNNF000000000	Gogerddan Farm, Aberystwyth	This study
AM007	*Corallococcus exiguus*	10.46	69.6	1264	8357	224	14471	29x	JABNNG000000000	Gogerddan Farm, Aberystwyth	This study
CA046D	*Corallococcus exiguus*	10.5	69.5	955	8310	122	26352	31x	JABNNE000000000	Carmarthen	[33]
AM011	*Myxococcus eversor*	11.62	68.9	688	9104	110	33389	82x	JABXEM000000000	Aberystwyth Harbour	This study
CA033	Myxococcus llanfairensis *	11.62	68.8	115	8928	9	312747	17x	JABUMU000000000	Tanerdy Woods, Carmarthen	[33]
CA039A	Myxococcus llanfairensis *	11.59	68.7	849	9039	133	27584	59x	JABUMQ000000000	Tanerdy Woods, Carmarthen	[33]
CA040A	Myxococcus llanfairensis *	11.72	68.9	70	8992	5	1036580	23x	JABUMR000000000	Tanerdy Woods, Carmarthen	[33]
CA051A	Myxococcus llanfairensis *	11.45	68.9	106	8765	7	562936	22x	JABUMS000000000	Llansteffan	[33]
CA056	Myxococcus llanfairensis *	11.36	68.9	77	8742	5	658787	26x	JABUMT000000000	Llansteffan	[33]
AM001	*Myxococcus vastator*	9.8	68.8	1946	8912	74	43403	115x	JABXEN000000000	Anglesey	This study
AM009	*Myxococcus vastator*	8.8	70	550	6926	61	45904	138x	JABXEP000000000	Clarach, Ceredigion	This study
AM010	*Myxococcus vastator*	8.93	70	276	6987	34	77358	125x	JABXEO000000000	Gogerddan Farm, Aberystwyth	This study
AB023	*Myxococcus xanthus*	9.13	68.9	221	7181	30	102234	144x	JABFNQ000000000	Gogerddan Farm, Aberystwyth	[33]
AM003	*Myxococcus xanthus*	9.14	69.2	380	7130	57	51156	109x	JABFNS000000000	Anglesey	This study
AM005	*Myxococcus xanthus*	9.15	69.2	413	7148	53	55319	148x	JABFNT000000000	Anglesey	This study
CA029	*Myxococcus xanthus*	9.19	68.8	584	7186	48	56679	93x	JABFNR000000000	Carmarthen	[33]
CA059B	*Pyxidicoccus fallax*	13.39	70.5	1321	10272	232	17466	25x	JABJTR000000000	Llansteffan	[33]

* *Myxococcus llanfairensis* is an abbreviation of *Myxococcus llanfairpwllgwyngyllgogerychwyrndrobwllllantysiliogogogochensis*.

## Supplementary Materials

The following are available online at www.mdpi.com/article/10.3390/microorganisms9102143/s1, Table S1: Myxobacterial genome sequences and MAGs in Genbank.

## Appendix A

The phylogenetic tree in Figure 2 was generated using the ‘one-click’ option on the phylogeny.fr webserver. A multiple sequence alignment was generated using MUSCLE and curated with Gblocks. The maximum likelihood tree was constructed using PhyML and rendered with TreeDyn, all with default parameters [139].
